# Evolutionary conservation analysis increases the colocalization of predicted exonic splicing enhancers in the *BRCA1 *gene with missense sequence changes and in-frame deletions, but not polymorphisms

**DOI:** 10.1186/bcr1324

**Published:** 2005-09-22

**Authors:** Christopher Pettigrew, Nicola Wayte, Paul K Lovelock, Sean V Tavtigian, Georgia Chenevix-Trench, Amanda B Spurdle, Melissa A Brown

**Affiliations:** 1 School of Molecular and Microbial Sciences, The University of Queensland, St. Lucia, Queensland, Australia; 2 Queensland Institute of Medical Research, Herston, Queensland, Australia; 3 International Agency for Research on Cancer, Lyon, France

## Abstract

**Introduction:**

Aberrant pre-mRNA splicing can be more detrimental to the function of a gene than changes in the length or nature of the encoded amino acid sequence. Although predicting the effects of changes in consensus 5' and 3' splice sites near intron:exon boundaries is relatively straightforward, predicting the possible effects of changes in exonic splicing enhancers (ESEs) remains a challenge.

**Methods:**

As an initial step toward determining which ESEs predicted by the web-based tool ESEfinder in the breast cancer susceptibility gene *BRCA1 *are likely to be functional, we have determined their evolutionary conservation and compared their location with known *BRCA1 *sequence variants.

**Results:**

Using the default settings of ESEfinder, we initially detected 669 potential ESEs in the coding region of the *BRCA1 *gene. Increasing the threshold score reduced the total number to 464, while taking into consideration the proximity to splice donor and acceptor sites reduced the number to 211. Approximately 11% of these ESEs (23/211) either are identical at the nucleotide level in human, primates, mouse, cow, dog and opossum *Brca1 *(conserved) or are detectable by ESEfinder in the same position in the *Brca1 *sequence (shared). The frequency of conserved and shared predicted ESEs between human and mouse is higher in *BRCA1 *exons (2.8 per 100 nucleotides) than in introns (0.6 per 100 nucleotides). Of conserved or shared putative ESEs, 61% (14/23) were predicted to be affected by sequence variants reported in the Breast Cancer Information Core database. Applying the filters described above increased the colocalization of predicted ESEs with missense changes, in-frame deletions and unclassified variants predicted to be deleterious to protein function, whereas they decreased the colocalization with known polymorphisms or unclassified variants predicted to be neutral.

**Conclusion:**

In this report we show that evolutionary conservation analysis may be used to improve the specificity of an ESE prediction tool. This is the first report on the prediction of the frequency and distribution of ESEs in the *BRCA1 *gene, and it is the first reported attempt to predict which ESEs are most likely to be functional and therefore which sequence variants in ESEs are most likely to be pathogenic.

## Introduction

Studies of the pathogenicity of nucleotide sequence variants in disease-associated genes usually focus on the effect on encoded protein structure and function. However, the deleterious effects of such changes may also be attributed to regulatory defects such as altered transcript stability [1] and abnormal transcript splicing [2-4].

Splicing is the process of removing introns from pre-mRNA transcripts. Splicing signals include a number of sequence elements in the precursor mRNA, including splice site (donor and acceptor) consensus sequences and a branch point sequence (for review, see Cartegni and coworkers [5]). Splicing is mediated by the spliceosome – a complex of small nuclear RNAs and associated proteins that recognizes splicing signals and catalyzes intron removal. The splice site consensus sequences and the sequences of small nuclear RNAs are highly conserved through evolution (for reviews, see Hastings and Krainer [6] and Black [7]). Disease-associated mutations in splicing consensus sequences have been identified in several genes. In *BRCA1*, for example, the IVS10-2A->C mutation [8] and the IVS20+1G>A mutation [9] are strongly associated with breast cancer predisposition.

Splicing is also regulated by exonic splicing silencers and exonic splicing enhancers (ESEs). ESEs, and the arginine/serine-rich family of proteins (SR proteins) that bind to these sequences (for review, see Cartegni and coworkers [5]) are conserved across vertebrates [10]. Recently, bioinformatic sequence analysis tools (ESEfinder and Rescue-ESE) that predict the presence of ESEs became available, and these may facilitate prediction of the effect of sequence variants on transcript splicing [10,11]. Mutations that lead to alterations in ESE motifs can result in failure of SR proteins to recognize and bind to the ESE motif, which in turn leads to failure of the spliceosome machinery to recognize the exon, resulting in exon skipping. A single nucleotide mutation in exon 18 of *BRCA1 *(G5199T), for example, has been shown to disrupt an ESE motif recognized by SF2/ASF, leading to exon skipping [2]. Single exonic nucleotide sequence variations resulting in altered mRNA transcript splicing have also been reported as contributing to other diseases, for example spinal muscular atrophy [12,13].

The *BRCA1 *gene is implicated in both familial and sporadic breast cancers. *BRCA1 *encodes a 1863 amino acid nuclear phosphoprotein with tumor suppressor activity, with functions that include the regulation of DNA damage repair, transcription, cell cycle arrest and apoptosis (for review, see Venkitaraman [14]). Loss of *BRCA1 *function is likely to result in genomic instability through a combination of loss of cell cycle checkpoint control post-DNA damage, loss of efficient DNA damage repair, and loss or limited initiation of apoptosis pathways. Loss of *BRCA1 *function can arise due to mutation and by aberrant regulation at the transcriptional and post-transcriptional levels [15,16].

Over a thousand *BRCA1 *single nucleotide sequence variations have been reported in the Breast Cancer Information Core (BIC) database [17]. Given that *BRCA1 *sequencing (and thus variant identification) is generally undertaken in individuals with cancer or with a family history of cancer, it is assumed that the majority of these variants were identified in such individuals, and that at least a fraction of them predispose to cancer. The pathogenicity of only a small number of variants has been tested functionally or inferred genetically. It is likely that a proportion of these sequence variants, including some missense mutations and apparent truncation variants such as G5199T [2], may exert a pathogenic effect by disrupting ESE motifs and altering *BRCA1 *splicing.

At least 30 alternatively spliced isoforms of *BRCA1 *have been identified, but only a small proportion of these – including the full length, Δ9–10, Δ11q and Δ9–10,11q isoforms – have been shown to be expressed in a wide variety of cell and tissue types (for review, see Orban and Olah [18]). A recently identified protein produced from the *BRCA1 *locus is BRCA1-IRIS, which arises via abnormal *BRCA1 *splicing [19]. The precise mechanisms that underlie the generation of normal and abnormal *BRCA1 *splice variants, and in particular the role of ESEs and SR proteins, are not well understood.

We set out to predict functional ESEs in *BRCA1 *using a multifaceted bioinformatics approach. The ESEfinder program was used to predict ESEs located proximally to the ends of exons in the human *BRCA1 *gene. The evolutionary conservation of these ESEs was then examined among primate, mouse, cow, dog and opossum *Brca1 *sequences. Reported sequence variations in *BRCA1 *were compared with predicted ESEs, as a means of identifying sequence changes that may exert pathogenic effects through altering the splicing of *BRCA1 *transcripts.

## Materials and methods

### Predicting exonic splice enhancers

The web-based ESEfinder 2.0 program [11,20] searches for sequences that act as binding sites for four members of the serine/arginine rich family of splicing enhancer proteins. Input sequences are screened for consensus binding sequences for the SR proteins SF2/ASF, SC35, SRp40 and SRp55, developed using the SELEX (systematic evolution of ligands by exponential enrichment) procedure [11,21,22]. The program scores the input sequences according to fit with the loose consensus sequences; scores above a default threshold value are predicted to act as SR protein binding sites and thus function as ESEs. Increased threshold values of 2.0 for SF2/ASF (from 1.956) and 3.0 for SC35 (from 2.383), SRp40 (from 2.670) and SRp55 (from 2.676) were used in order to minimize false-positive results [23]. The wild-type *BRCA1 *cDNA sequence (GenBank:U14680) was used as input in an exon-by-exon manner. The *BRCA1 *open reading frame (ORF) begins 19 nucleotides into exon 2 and continues 125 nucleotides into exon 24. The alternative first exons 1a and 1b were not analyzed because it was assumed that the inclusion of these exclusive exons was driven by promoter use and not splicing factors. *BRCA1 *sequences for introns 5, 6, 9 and 10 (GenBank:L78833) were also screened in their entirety using ESEfinder under the same conditions.

### Cross-species sequence analysis

The *Brca1 *cDNA sequences for a selection of four primate organisms ('the primates': chimpanzee [*Pan troglodytes*, GenBank:AY365046], orang-utan [*Pongo pygmaeus*, GenBank:AY589040], rhesus monkey [*Macaca mulatto*, GenBank:AY589041] and gorilla [*Gorilla gorilla*, GenBank:AY589042]), mouse (*Mus musculus*, GenBank:NM_009764), Cow (*Bos Taurus*, GenBank:NM_178573), opossum (*Monodelphis domestica*, GenBank:AY994160) and dog (*Canis familiaris*, Genbank:NM_001013416) were obtained from the National Center for Biotechnology Information [24]. Splice junctions were predicted by aligning sequences to the human *BRCA1 *exon sequences. The *Brca1 *cDNA sequences were then screened using ESEfinder 2.0 in an exon-by-exon manner using the same threshold scores as were used for the human sequence. Nucleotide sequence alignments were performed using a clustal alignment program, with gaps between sequences noted and accounted for when determining position of predicted ESEs within exons. Mouse *Brca1 *sequences for introns 5, 6, 9 and 10 (chr11:101160890-101222966) were obtained from the University of California Santa Cruz Genome Bioinformatics project [25].

### Previously reported *BRCA1* sequence variants

The online BIC database [17] contains detailed information on sequence variants in *BRCA1*. The BIC database was used as resource for a range of mutation types analyzed, including missense (*n *= 17), nonsense (*n *= 167), splicing (*n *= 5), insertion or deletion mutations that maintained the ORF (*n *= 19), unclassified variant (UV; *n *= 387) and polymorphisms (*n *= 12). Mutations were examined only if they were located within an exon. Each variant cDNA sequence was then screened using ESEfinder 2.0 in an exon-by-exon, variant-by-variant manner, and the results were compared with those for wild-type sequence to identify any loss, gain, or alteration in predicted SR protein binding sites.

For the colocalization analysis, additional single nucleotide polymorphisms (SNPs) were obtained from the SNPper Gene Finder online database [26] (*BRCA1 *SNP data set SS965). Only exonic SNPs were analyzed, and those also reported in the BIC database were not included. In total, 21 SNPs from SNPper and 12 from BIC were used in this analysis.

### Amino acid substitution analysis

An alignment of 12 full-length BRCA1 protein sequences was made using the multiple sequence alignment program 3DCoffee [27], which also incorporates alignment to X-ray and nuclear magnetic resonance (NMR) structures. The program was run using Mlalign_id_pair, Mslow_pair and Mclustalw_aln to generate amino acid alignments, and Mfugue_pair to generate structure sequence alignments. GenBank accession numbers for BRCA1 protein sequences used in the alignment were as follows: human (*Homo sapiens*), NP_009225; chimpanzee (*Pan troglodytes*), AAG43492; gorilla (*Gorilla gorilla*), AAT44835; orang-utan (*Pongo pygmaeus*), AAT44834; rhesus macaque (*Macaca mulatta*), AAT44833; mouse (*Mus musculus*), AAD00168; dog (*Canis familiaris*), AAC48663; cow (*Bos taurus*), NP_848668; gray, short-tailed opossum (*Monodelphis domestica*), AY994160; chicken (*Gallus gallus*), NP_989500; African clawed frog (*Xenopus laevis*), AAL13037; and green-spotted pufferfish (*Tetraodon nigroviridis*), AAR89523. For the structure component of the alignment, we used BRCA1 RING NMR structure 1JM7.pdb and the BRCA1 BRCT repeat crystal structures 1JNX.pdb and 1T29.pdb [28-30].

BRCA1 missense substitutions were analyzed against the alignment using two programs, namely SIFT and A-GVGD [31,32]. The joint analysis was used to define a group of substitutions that should be highly enriched for deleterious mutations and a group that should be highly enriched for neutral substitutions. The enriched deleterious group consisted of those substitutions that had SIFT scores ≤0.05 and A-GVGD scores of GV<62 and GD>0. The enriched neutral group consisted of those substitutions that had SIFT scores >0.1 and A-GVGD scores of GV>0 and GD = 0.

### Rescue-ESE

A limited analysis of *BRCA1 *(exons 2–10) was also performed using the recently described ESE detection program Rescue-ESE [10,33] using the web-based facility [34]. Human *BRCA1 *was analyzed using the human algorithm whereas mouse *Brca1 *was analyzed using the mouse algorithm.

### Statistical analyses

Differences in the frequency of ESEs in exons and introns, and in the colocalizations of ESEs with reported sequence changes were assessed using the Pearson χ^2 ^test. When expected cell numbers were smaller than five, the Fisher's exact test rather than Pearson χ^2 ^test was used to obtain an estimate of significance.

## Results

### *BRCA1 *exons contain numerous putative exonic splice enhancer sequences

ESEfinder initially predicted 669 ESEs in the 5592 nucleotide *BRCA1 *ORF. In order to reduce the number of potential false positives, increased threshold scores were used, in accordance with the approach used to study ESEs in *TP53 *[23]. After increasing the threshold score to 2.0 for SF2/ASF and 3.0 for SC35, SRp40 and SRp55, ESEfinder predicted 464 ESEs in *BRCA1 *(Table 1). The majority of these potential ESEs are predicted to bind SRp40 and SF2/ASF (162 and 145 ESEs, respectively), whereas smaller numbers are predicted for SC35 and SRp55 (92 and 65, respectively).

*BRCA1 *exon 11 comprises over 60% of the coding sequence, and 57% (265/464) of ESEfinder predicted ESEs were located in the exon. Although some regulatory elements can be located several kilobases away [35], studies of the molecular mechanisms of splicing suggest that ESEs occupy specific positions relative to the 5' and 3' ends of exons [13,36]. Thus, it is less likely that predicted ESEs in the centre of *BRCA1 *exon 11 are functional. We therefore employed an exon 'cutoff' for our analysis that limits the amount of exon sequence analyzed to the first and last 125 nucleotides of an exon. The introduction of the nucleotide limit affects only exons 11 (3.4 kilobases in length) and exon 16 (311 nucleotides in length), and reduces the total number of predicted ESEs to 211.

### Approximately 11% of predicted *BRCA1 *exonic splice enhancers are evolutionarily conserved

**Figure 1:**
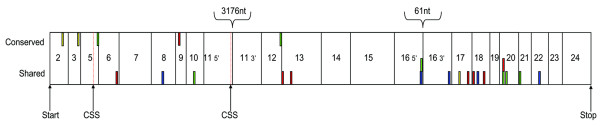
Shared and conserved predicted ESEs in the human and mouse *BRCA1 *genes. The *BRCA1 *open reading frame (ORF) divided into exons is shown to scale, with shorter analyzed fragments of exons 11 and 16. Conserved exonic splicing enhancers (ESEs) are shown on the top of the ORF, with shared ESEs shown on the bottom. Overlapping ESE motifs are staggered. SF2/ASF ESE motifs are shown in red, with SC35 in blue, SRp40 in green and SRp55 in yellow, also to scale. Cryptic splice sites (CSSs) in exons 5 and 11 are marked. Only the sequence within 125 nucleotides (nt) of exonic splice donor and acceptor consensus sequences has been analysed. Conserved ESEs are those sequences that were identical in human, primates, mouse cow, dog and opossum *Brca1*. Shared ESEs are those sequences for which ESEfinder predicted a binding site for the same arginine-serine rich protein (SR protein) to the same exon position but the motif sequence was not the same between species.

The molecular mechanisms that underlie the major splicing processes are highly conserved through evolution (reviews available elsewhere [5,7,37]). Given this and the high nucleotide sequence conservation of ESEs [10] and amino acid homology of the SR proteins that bind ESEs (e.g. see Hanamura and coworkers [38]), it is reasonable to suggest that those ESEs that are also found in *Brca1 *sequences from other species are more likely to be functional. The *BRCA1 *mRNA sequences from human, four primates as well as mouse, cow, dog and opossum were analyzed. Gaps in sequence alignments were accounted for, and ESEfinder 2.0 outputs were compared with that for the human sequence. Output was then divided in two: 'conserved ESEs', referring to those predicted ESEs that were identical in sequence and exon position across all species examined; and 'shared ESEs', referring to those sequences for which ESEfinder predicted a binding site for the same SR protein to the same exon position, although the nucleotide sequence of the motif was not identical across species (Fig. 1). In approximately 2% (5/211) of predictions, identical ESE consensus sequences predicted in the human *BRCA1 *sequence were also found in all *Brca1 *sequences examined. In a further 9% (18/211) of cases the same ESE was predicted in the same position of human *BRCA1 *and other *Brca1 *sequences, albeit with a different nucleotide sequence. Together, these findings suggest that approximately 11% (23/211) of ESEs are likely to be present in human *BRCA1*, primate, mouse, cow, dog and opossum *Brca1 *genes.

### Conserved exonic splice enhancers are more frequent in *BRCA1 *exons than introns

The frequency of predicted ESE motifs in exons averages 11.96 ESEs per 100 nucleotides for complete exonic sequence, which is greater than the number of predicted ESEs in a random sample of *BRCA1 *intronic DNA (introns 5, 6, 9 and 10), which occur at a rate of 10.90 per 100 nucleotides. Given that SR proteins primarily bind to sequences in exonic DNA [5], this suggests that there is potentially a high level of false positives associated with ESEfinder.

Applying the increased threshold decreased the frequency of predicted ESEs in both exons (8.30 predicted ESEs per 100 nucleotides) and introns (7.66 predicted ESEs per 100 nucleotides), with no significant difference. However, applying the 125 nucleotide cutoff changed the frequency in exons to 8.96 predicted ESEs per 100 nucleotides and in introns to 5.6 predicted ESEs per 100 nucleotides (*P *= 0.001). This suggests that applying the 125 nucleotide cutoff does indeed increase the specificity of ESEfinder output.

To investigate whether evolutionary analysis was able to further filter out false positives detected by ESEfinder, regions of human and mouse intronic sequences were also analyzed using ESEfinder. Again, the 125 nucleotide cutoff limit was also used in this analysis. Shared and conserved motifs between human and mouse introns were found to occur at a frequency of 2.845 per 100 nucleotides of exonic sequence, compared to 0.6 per 100 nucleotides of intronic DNA (*P *= 0.00005). This suggests that evolutionary conservation together with the 125 nucleotide cutoff is likely to improve significantly the specificity of the ESEfinder output.

### Known *BRCA1 *sequence changes map to predicted exonic splice enhancers

The positions of ESEs predicted using the increased threshold for the full wild-type *BRCA1 *coding sequence were compared with the locations of *BRCA1 *sequence variants reported to the BIC database (Table 2). A total of 607 reported sequence variants in the *BRCA1 *ORF were examined, the majority (387) being UVs. Nonsense mutations were included in the analysis because such changes have previously been shown to induce splicing defects [2]. Of the sequence variants analyzed, 268 are located within predicted ESEs. These variants either result in loss of a potential ESE (*n *= 182), reduced score for an ESE (*n *= 48), or an increased score (*n *= 38). A further 93 sequence variations analyzed were predicted to result in the gain of one or more potential ESEs. A total of 59% (361/607) of the sequence changes analyzed alter the ESEfinder predictions for the complete *BRCA1 *ORF.

### Of the *BRCA1 *sequence variants reported on BIC, 22 colocalize with evolutionarily conserved exonic splice enhancers

Data from the ESE conservation analysis (with the 125 nucleotide cutoff) was then compared with the *BRCA1 *sequence variants reported on the BIC database. This analysis identified 22 sequence variants colocalizing with shared/conserved ESEs located within 125 nucleotides of exon junctions (Table 3), with a total of 14/23 (61%) of the predicted ESEs affected by these sequence changes (Table 4). Importantly, these sequence changes include the G5199T change in *BRCA1 *exon 18 that has previously been shown to affect ESE-mediated splicing [2]. Interestingly, nine of the sequence changes that localize to this subset of predicted ESEs are currently classified as UVs.

### Appying filters to the exonic splice enhancer prediction increases the colocalization with reported missense and insertion–deletion changes

To assess whether the prioritization process is predicting *BRCA1 *ESEs that are more likely to be targeted by sequence changes, we determined the percentage colocalization of predicted ESEs with reported sequence changes in BIC before and after increasing the ESEfinder threshold, considering the location relative to exon ends and taking into account evolutionary sequence conservation (Table 4). Without any filtering of ESE prediction, we found that 51.72% of predicted ESE motifs colocalized with reported sequence variations. After the full filtering process, in which the increased threshold and 125 nucleotide limits were used along with full evolutionary analysis, we found that 60.87% of predicted ESEs colocalized with reported sequence variations. Assuming that the filtering process has enhanced the prediction of functional ESEs in *BRCA1*, this suggests that at least a proportion of the variants located within them may result in altered ESE function.

The effect of applying these filters on colocalization with the different types of *BRCA1 *sequence variants was then determined. We hypothesized that if the filtering process was effective, then the colocalization with sequence changes most likely to be disease associated would increase, whereas the colocalization with known polymorphisms would decrease. Consistent with this hypothesis, the filtering process always increased the percentage of ESEs colocalizing with possible disease-associated changes (Table 4). This was especially marked for missense changes, in which the increase was over 400% (*P *= 0.07), but was also observed for in-frame deletions, with an increase of 53% (*P *= 0.4). In contrast, colocalization with known polymorphisms decreased by 100% (*P *= 0.6).

In the analysis of colocalization with UVs, we first categorized them according to their predicted affect on protein function. One of the rationales for this stems from the previous report that 'conservative' amino acid sequence changes in *hMLH1 *and *hMSH2 *genes colocalize with predicted ESEs, whereas 'radical' changes exhibit no association [39]. The majority of UV sequence changes were predicted to be neutral (174/254), which is consistent with the fact that a large proportion of UVs are expected to be benign [31]. For deleterious UV sequence changes, an increase in colocalization was observed (+206%), which was in accordance with observations for possible disease-associated changes. In contrast, neutral UV sequence changes exhibited a decrease in colocalisation (-53.8%), similar to that observed for polymorphisms.

### Rescue-ESE identifies a partially overlapping set of predicted exonic splice enhancers

An alternative ESE prediction program, namely 'Rescue-ESE', has recently become available [10,33]. This program predicts the presence of ESE motifs based on a list of 238 hexamer motifs found to be significantly enriched in exonic sequence, when compared with intronic sequence, and significantly enriched in exons with weak splice signals compared with exons with strong splice signals. In the Rescue-ESE program, no account is taken of the known recognition sites of splicing factors. A preliminary analysis of exons 2–10 of the *BRCA1 *gene using Rescue-ESE identified 116 potential ESEs, as compared with 47 detected in the same region by ESEfinder (Table 5). Strikingly, only 11 of the ESEs predicted by both programs (23% of ESEs predicted by ESEfinder) were the same. When evolutionary sequence conservation (with primates and mouse only for ESEfinder, and with mouse only for Rescue-ESE) was taken into account, however, the proportion of ESEfinder predicted ESEs that were also detected by Rescue-ESE increased to 45%. Together these findings support the notion that evolutionary conservation analysis increases the specificity of the current ESE prediction programs and emphasizes the need for both improved algorithms and biochemical validation of results. However, it should be noted that Rescue-ESE can predict ESEs recognized by SR proteins other than those used by ESEfinder.

## Discussion

In this study we attempted to identify ESEs in *BRCA1 *and to predict which are most likely to be functional. As one of the ultimate purposes of the study was to determine the *trans*-acting factors that are involved in splicing decisions of *BRCA1*, our analysis principally focused on the ESE detection program ESEfinder. Initial screens using this program generated a large number of predicted ESEs in the *BRCA1 *gene. Evolutionary conservation analysis reduced the number dramatically. The observation that conserved ESEs are much more frequent in exons than in introns (4.67:1 versus 1.10:1 for all ESEs) is consistent with the fact that SR proteins primarily recognize exonic sequences and suggests that evolutionary conservation analysis, together with applying the 125 nucleotide cutoff, can reduce the number of false positives significantly. The low frequency of conserved ESEs in introns may represent intronic splicing enhancer sequences that bind to SR proteins or may represent background noise in the prediction program. Although intronic splicing enhancers are best known for their association with the spliceosome [40,41], it is also known that SR proteins can bind to intronic splicing elements [42].

The increased threshold used in the ESEfinder analysis was designed to reduce the number of potential false-positive results. Increased thresholds have been used in bioinformatics analysis previously with other cancer-related genes, including *hMSH1 *and *hMLH2 *[39], and *TP53 *[23]. In their earlier report, Gorlov and coworkers [39] employed an increased threshold of 3.0 for SF2/ASF in preference to the recommended 1.956. In their more recent report [23], the threshold score used for analysis of *TP53 *for SF2/ASF was 2.0. Liu and coworkers [2] previously identified and biochemically analyzed an ESE motif recognized by SF2/ASF in exon 18 of *BRCA1*, with an ESEfinder score of 2.143. Therefore, had the increased threshold score of 3.0 been used for SF2/ASF in this study, then at least one confirmed ESE would have been missed. The creators of ESEfinder also emphasize that there is no strict quantitative correlation between numerical scores and ESE activity [11], particularly because ESEs are dependent on other important variables such as splice site strength, local sequence context and the presence of other splicing *cis*-elements.

ESEs are often found grouped in areas within exons [43]. Therefore as expected, many predicted ESEs overlapped partially or entirely with other predicted ESEs for identical or different SR proteins. Consequently, the amount of *BRCA1 *coding sequence covered by the 464 predicted ESEs is only 2388 nucleotides, which is 42% of the ORF. This is lower than the 57% of sequence covered by predicted ESEs for another tumor suppressor gene, namely *TP53 *[23]. Interestingly, only two natural alternatively spliced isoforms of *TP53 *have been found [44,45], compared with the four predominant isoforms of *BRCA1 *(for review, see Orban and Olah [18]).

*BRCA1 *produces four predominant splicing isoforms – the full length, Δ9–10, Δ9–10,11q and Δ11q isoforms – at varying levels in a range of cell and tissue types [46,47] (for review, see Orban and Olah [18]). Two of these predominant variants (Δ11q and Δ9–10,11q) employ a cryptic splice site located 118 basepairs into exon 11, and when used lead to a deletion of over 3 kilobases of the exon. We excluded the majority of exon 11 by limiting the analysis, looking at only the first 125 and final 125 nucleotides of the exon. No conserved or shared ESEs were located in these regions of exon 11, but this analysis may have missed potentially active sequences. Given that exon 11 is unusually large for an exon, it is possible that splicing regulatory elements are active throughout the exon to ensure accurate splicing. Fewer ESEs in proximity to the splice site required for full length exon 11 may contribute to a less efficient recognition of the splice site by the spliceosome, and as a consequence the spliceosome may have a higher affinity for the upstream cryptic splice site. Currently, only the Δ11 *Brca1 *splice variant has been confirmed to be present in mouse [48,49], but no comprehensive search results have been reported for alternatively spliced *Brca1 *transcripts. Interestingly, during cloning of the bovine *Brca1 *cDNA, the alternatively spliced transcripts Δ11 and Δ11b were detected in spleen RNA [50].

The association of *BRCA1 *sequence variants with predicted ESE motifs before incorporation of evolutionary data varies between exons. Exon 5 contains a high percentage of sequence variants located in predicted ESEs, and has previously been shown to be alternatively spliced [3,51-53]. Some of these alternatively spliced transcripts arise due to sequence mutations, whereas others appear to be expressed normally. The high number of predicted ESEs associated with sequence variants in exon 5 may indicate that these ESEs play functional roles in *BRCA1 *splicing processes.

Comparing prioritized predicted ESEs with known sequence variants in *BRCA1 *highlights 14 reported sequence changes that are predicted to affect ESEs (Table 3). Importantly, the G1599T mutation that has previously been shown to affect ESE-mediated splicing of *BRCA1 *[2] was included in this group. Nine (50%) of the sequence changes affecting conserved or shared predicted ESEs are currently designated as UVs. Although genetic and cellular studies are currently ongoing to classify such variants [54-56], the fact that they map to these conserved ESEs suggests that RNA splicing analyses (e.g. Tesoriero and coworkers [9]) should be included in the list of assays used to investigate the consequences of these sequence changes.

## Conclusion

**Figure 2:**
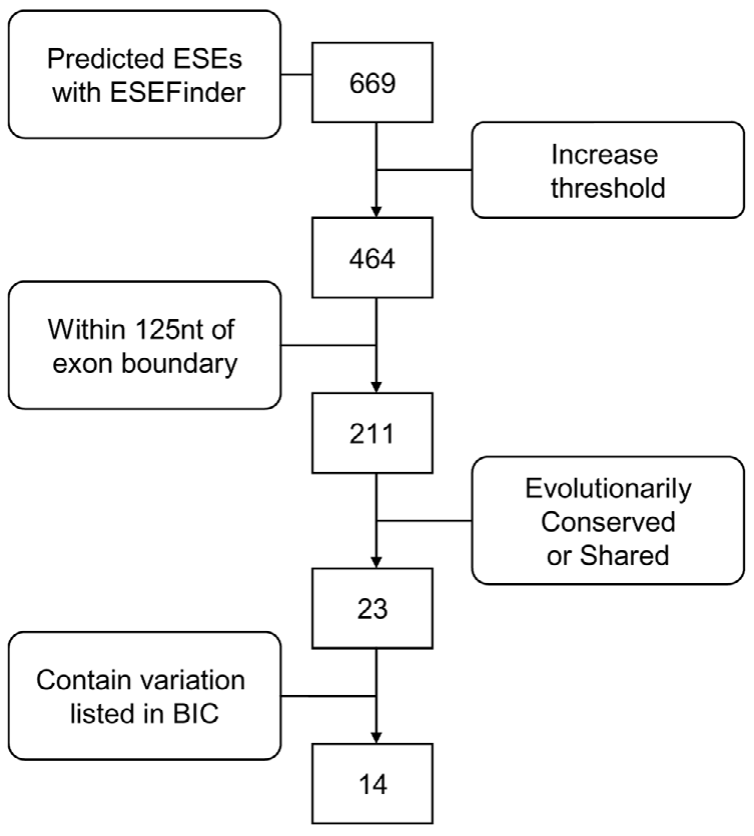
Prioritizing ESEs in the coding region of *BRCA1*. Shown is a flow diagram of the process of prioritizing exonic splicing enhancers (ESEs) in the *BRCA1 *open reading frame for biochemical analysis. Numbers indicate predicted ESEs following each stage.

This *in silico *study has prioritized a select group of predicted ESEs for functional analysis. These ESEs may be critical in regulating the alternative splicing patterns of *BRCA1*, and disruption of alternative splicing may have important implications for breast tumorigenesis. We show that a combination of threshold score, position relative to exon:intron boundaries and evolutionary conservation may significantly improve the specificity of the ESEfinder output (Table 4, Fig. 2). The study also classified a subset of *BRCA1 *UVs as potentially affecting *BRCA1 *splicing via disruption of predicted ESEs. It is also possible that there remains a number of mutations in the BIC database that have been classified incorrectly, such as G5199T, which is currently listed as a nonsense mutation, although it has been shown to alter *BRCA1 *splicing [2]. The data generated in the present study may be considered useful but not definitive for the assignment of pathogenicity of sequence variants. Indeed, comparing ESEfinder with Rescue-ESE (Table 5) highlights the need for additional complementary analyses such as evolutionary conservation. Ultimately, however, functional analysis of these prioritized predicted ESEs and corresponding sequence changes will be essential to validate the *in silico *approach used in this and other studies.

## Abbreviations

BIC = Breast Cancer Information Core; ESE = exonic splicing enhancer; ORF = open reading frame; NMR = nuclear magnetic resonance; SNP = single nucleotide polymorphism; SR protein = arginine-serine rich protein; UV = unclassified variant.

## Competing interests

The authors declare that they have no competing interests.

## Authors' contributions

CP conducted ESEfinder analysis, BIC database analysis, evolutionary conservation analyses, analysis and interpretation of data, day-to-day running of project, drafting and redrafting of the manuscript. NW conducted evolutionary conservation analysis. PKL established the conditions for analyzing *BRCA1* with ESEfinder, provided ongoing advice on the use of the program and help in interpreting data; he also critically read the manuscript. SVT provided the opossum *Brca1* sequence, analyzed the effect of UV sequence change on protein, and critically read the manuscript. GC-T suggested determining relative evolutionary conservation on ESEs predicted in introns versus exons, suggested a preliminary analysis of *BRCA1* with Rescue-ESE, and critically read the manuscript. ABS suggested Fig. 2, conducted statistical analysis, and critically read the manuscript. MAB conceived and managed the project and made major contributions to interpreting the results, suggesting additional data required, and writing the manuscript.

## Figures and Tables

**Table 1:** Putative ESEs in *BRCA1*

Exon	Length (nt)	Predicted SF2/ASF ESEs	Predicted SC35 ESEs	Predicted SRp40 ESEs	Predicted SRp55 ESEs	Total predicted ESEs	Nucleotides mapping to predicted ESEs	ESEs as % of exon
2	80	0	0	1	1	2	13	16.250
3	54	0	0	2	1	3	18	33.333
5	78	2	0	4	2	8	41	52.564
6	89	1	0	3	0	4	22	24.719
7	140	2	3	4	3	12	57	40.714
8	106	4	3	3	1	11	51	48.113
9	46	1	0	0	0	1	7	15.217
10	77	2	1	3	0	6	28	36.364
11	3426 (250)	90 (7)	48 (4)	93 (7)	34 (2)	265 (20)	1405 (106)	41.010 (42.400)
12	89	3	1	2	2	8	44	49.438
13	172	4	3	4	3	14	80	46.512
14	127	4	2	7	2	15	66	51.969
15	191	7	2	6	3	18	105	54.974
16	311 (250)	5 (4)	9 (6)	10 (8)	3 (1)	27 (19)	143 (110)	45.981 (44.000)
17	88	3	4	2	2	11	49	55.682
18	78	3	1	1	1	6	32	41.026
19	41	0	0	0	0	0	0	0.000
20	84	2	4	4	0	10	38	45.238
21	55	1	1	2	0	4	23	41.818
22	74	2	1	2	3	8	40	54.054
23	61	3	3	2	1	9	37	60.656
24	125	6	6	7	3	22	89	71.200
								
Total	5592 (2355)	145 (61)	92 (45)	162 (74)	65 (31)	464 (211)	2388 (1056)	42.704 (44.841)

Numbers in parentheses refer to those sequences within 125 nucleotides (nt) of the exon end. ESE, exonic splicing enhancer.

**Table 2:** Known *BRCA1 *sequence changes affecting potential ESEs in the *BRCA1 *open reading frame

Type	Number examined	Sequence changes resulting in loss of ESE	Sequence changes resulting in altered score of ESE	Sequence changes resulting in gain of ESE motif	Total sequence changes affecting putative ESEs (%)
MS	17	5	7	1	13 (76.471)
UV	387^a^	104	57	68	229 (59.173)
IFD/I	19	8	3	2	13 (68.421)
P	12	3	2	2	7 (58.333)
NS	167	62	17	20	99 (59.281)
S	5	0	0	0	0 (0.000%)
Total	607	182	86	93	361 (59.473)

^a^One mutation listing in the Breast Cancer Information Core database as unclassified variant (UV) encodes a nonsense mutation. There are 386 missense UVs. ESE, exonic splicing enhancer; IFD/I, in-frame deletion/insertion; MS, missense; NS, nonsense; P, polymorphism; S, change to splicing consensus sequence.

**Table 3:** *BRCA1 *sequence variants mapping to shared or conserved ESEs

Exon	Predicted ESE	ESE location in exon	Sequence change	Class^a^	Number of times reported	Shared or conserved^b^	Affect on ESE^c^
2	SRp55	53	T172C	M-UV	3	Conserved	Decrease
3	SRp55	41	A243G	M-UV	3	Conserved	Increase
5	SRp40	72	A330G	M	16	Conserved	Increase
6	SF2/ASF	76	G411C	M-UV	1	Shared	Increase
8	SC35	47	A609C	M-UV	1	Shared	Increase
12	SRp40	82	C4302T	NS	26	Conserved	Lose
13	SF2/ASF	1	C4305T	NS	2	Shared	Lose
16	SC35	113	4917del15	IFD	1	Shared	Lose
17	SF2/ASF	68	C5173T	M-UV	1	Shared	Lose^d^
18	SF2/ASF	4	G5199T	NS	10	Shared	Lose^d^
18	SC35	22	G5215T	M	1	Shared	Lose
			G5215A	M	2		Decrease
20	SRp40	14	T5326C	M-UV	6	Shared	Decrease
			A5328T	NS	1		Decrease^d^
			G5331A	M-UV	4		Decrease^d^
			G5332A	M-UV	2		Lose^d^
			5332del3	IFD	2		Lose^d^
20	SF2/ASF	15	A5328T	NS	1	Shared	Decrease^d^
			G5331A	M-UV	4		Decrease^d^
			G5332A	M-UV	2		Decrease^d^
			5332del3	IFD	2		Lose^d^
20	SRp40	29	T5341G	M-UV	3	Shared	Lose

^a^Classification in Breast Cancer Information Core database: IFD, in-frame deletion; M, missense; M-UV, missense unclassified sequence variant; NS, nonsense. ^b^Exonic splicing enhancers (ESEs) shared or conserved between human, primates, mouse, cow, dog and opossum. ^c^Lose, loss of predicted ESE motif; increase, retain ESE motif with higher score; decrease, retain ESE motif with lower score. ^d^Alter additional other ESE motifs.

**Table 4:** Effect of filters on colocalization of predicted ESEs with reported sequence changes in the *BRCA1 *open reading frame

	No filters	Complete filters^a^	Percentage change from no filters to complete filters
Number of predicted ESEs	669	23	-
Percentage of predicted ESEs affected by sequence changes (% [*n*])	51.72% (346)	60.87% (14)	+17%
Percentage of predicted ESEs affected by NS^b ^sequence changes (% [*n*])	20.63% (138)	21.74% (3)	+5%
Percentage of predicted ESEs affected by IFD^b ^sequence changes (% [*n*])	8.52% (57)	13.04% (3)	+53%
Percentage of predicted ESEs affected by M^b ^sequence changes (% [*n*])	1.64% (11)	8.70% (2)	+430%
Percentage of predicted ESEs affected by UV^b ^sequence changes predicted to be deleterious^c ^(% [*n*])	8.52% (57)	26.10% (6)	+206%
Percent of predicted ESEs affected by UV^b ^sequence changes predicted to be neutral^c ^(% [*n*])	18.83% (126)	8.70% (2)	-54%
Percent of predicted ESEs affected by P^b,d ^sequence changes (% [*n*])	4.19% (28)	0.00% (0)	-100%

^a^Increased threshold, location within 125 nucleotides of exon end and conserved in human, primate, mouse, cow, dog and opossum. ^b^Classification in Breast Cancer Information Core (BIC) database: IFD, in-frame deletion; M, missense; M-UV, missense unclassified sequence variant; NS, nonsense. ^c^Deleterious refers to amino acid sequence changes predicted most likely to affect protein structure and function. ^d^P, polymorphisms include those reported in BIC and SNPper. ESE, exonic splicing enhancer.

**Table 5:** Comparison of ESEfinder and Rescue-ESE programs for identification of potential ESEs in exons 2–10 of the *BRCA1 *gene

	ESEs predicted by ESEfinder	ESEs predicted by Rescue-ESE	ESEs predicted by both programs	Proportion of ESEfinder predicted ESEs also detected by Rescue-ESE
*BRCA1 *exons 2–10 (total)	47	116	11	23%
*BRCA1 *exons 2–10 (conserved or shared ESEs between human and mouse)	20	69	9	45%
*BRCA1 *exons 2–10 (conserved or shared ESEs between human and mouse affected by breast cancer associated sequence changes)	16	16	3	19%
